# *In vivo* formation of natural HgSe nanoparticles in the liver and brain of pilot whales

**DOI:** 10.1038/srep34361

**Published:** 2016-09-28

**Authors:** Zuzana Gajdosechova, Mohammed M. Lawan, Dagmar S. Urgast, Andrea Raab, Kirk G. Scheckel, Enzo Lombi, Peter M. Kopittke, Katrin Loeschner, Erik H. Larsen, Glenn Woods, Andrew Brownlow, Fiona L. Read, Jörg Feldmann, Eva M. Krupp

**Affiliations:** 1 Trace Element Speciation Laboratory, Department of Chemistry, Meston Walk, University of Aberdeen, Aberdeen, AB24 3UE, UK; 2 United States Environmental Protection Agency, National Risk Management Research Laboratory, 5995 Center Hill Avenue, Cincinnati, OH 45224, USA; 3 Future Industries Institute, University of South Australia, Building X, Mawson Lakes Campus, Mawson Lakes, 5095 South Australia; 4 School Agriculture and Food Sciences, The University of Queensland, St Lucia, Queensland, 4072, Australia; 5 National Food Institute, Technical University of Denmark, Mørkhøj Bygade 19, DK-2860 Søborg, Denmark; 6 Agilent Technologies Ltd, 5500 Lakeside, Cheadle, SK8 3GR, UK; 7 SAC Wildlife Unit, Inverness, UK; 8 Oceanlab, University of Aberdeen, Main Street, Newburgh, Aberdeenshire, AB41 6AA, UK

## Abstract

To understand the biochemistry of methylmercury (MeHg) that leads to the formation of mercury-selenium (Hg-Se) clusters is a long outstanding challenge that promises to deepen our knowledge of MeHg detoxification and the role Se plays in this process. Here, we show that mercury selenide (HgSe) nanoparticles in the liver and brain of long-finned pilot whales are attached to Se-rich structures and possibly act as a nucleation point for the formation of large Se-Hg clusters, which can grow with age to over 5 μm in size. The detoxification mechanism is fully developed from the early age of the animals, with particulate Hg found already in juvenile tissues. As a consequence of MeHg detoxification, Se-methionine, the selenium pool in the system is depleted in the efforts to maintain essential levels of Se-cysteine. This study provides evidence of so far unreported depletion of the bioavailable Se pool, a plausible driving mechanism of demonstrated neurotoxic effects of MeHg in the organism affected by its high dietary intake.

Irreversible neurological damage to the central nervous system associated with exposure to methylmercury (MeHg) has led to strict regulations of MeHg concentrations in the food we consume^1^,^2^. The damage caused by MeHg is particularly dangerous during embryotic development as MeHg can be transported through the placenta to the foetus^3^,^4^. Prenatal exposure to MeHg has been linked with cognitive deficit in children^4^ and spontaneous abortions and stillbirth in severe cases^5^. At the cellular level, methylmercury-related toxic effects are thought to be caused by binding of MeHg to cysteinyl groups of proteins^6^, which may have severe implications on the synthesis of cellular glutathione, thus leading to oxidative damage. However, marine mammals and seabirds, which can accumulate unparalleled quantities of mercury (Hg) in the liver tissue do not present the toxic effects observed in humans and are able to tolerate brain-MeHg concentrations above the human threshold of severe neurological damage without showing apparent symptoms of poisoning^7^.

The protective effect of selenium (Se) against the toxicity of Hg species has long been observed and extensively studied under controlled conditions in exposure experiments^8^,^9^,^10^. Its pivotal role in detoxification is based on the high affinity of Hg to nucleophilic bio-selenols and subsequent formation of organic and inorganic compounds, including bio-mineralized mercury selenide (HgSe) particles. Although predominantly formed in the liver, HgSe particles were found in a wide range of biological tissues of whales and dolphins^11^,^12^ suggesting that the formation of HgSe is not a liver-specific process but takes place locally, in individual organs. Formation of HgSe particles is generally viewed as a detoxification mechanism, and an increasing number of authors argue that this mechanism may also have negative consequences on the biosynthesis of essential amino acids^13^. Selenium is covalently bound to Se-proteins as constituent of the 21^st^ amino acid selenocysteine (Se-cysteine), and Se-cysteine forms an essential part of the active centre in the respective Se-enzymes^14^. Amongst the wide variety of Se-protein’s metabolic roles, they are exceptionally efficient redox catalysts due to a completely ionized Se-cysteine moiety at physiological pH^13^. Thus, Se-proteins are able to neutralise and restore the cellular antioxidant defence system disrupted by oxidative stress due to displacement of redox-active centres in the proteins by heavy metals. However, formation of Hg-Se compounds may also disrupt normal biological cycles of Se-proteins and suppress its antioxidant activity. Additionally, deposition of HgSe particles could lead to reduced Se bioavailability and subsequent deficiency, which predisposes to a variety of biological pathologies including epilepsy^15^, coordination disorder^16^, autoimmune and infectious diseases^17^.

To address these issues, we investigated *in vivo* formation of natural HgSe particles in a pod of stranded long-finned pilot whales (*Globicephala melas*), with age of the animal and the potency of the environmentally relevant dose of dietary MeHg to disrupt the Se-proteins synthesis. This has not been previously investigated despite the substantial indications of the interaction between Hg and Se, and therefore we conducted a multi-method analytical approach on brain and liver samples of the stranded whales.

## Results

### Bioaccumulation of Hg and Se in liver and brain of pilot whales

**Figure 1:**
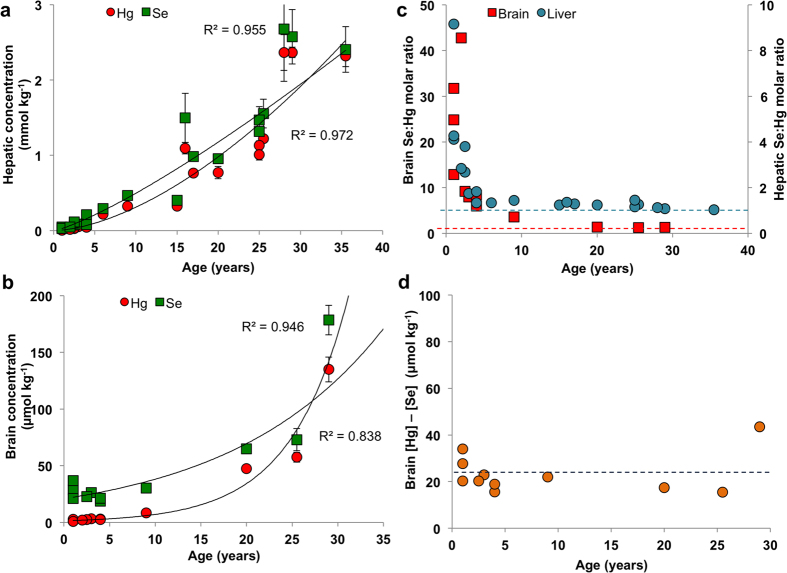
Bioaccumulation of Se and Hg in the liver (n = 21) and brain (n = 11) of stranded long-finned pilot whales (*Globicephala melas*). Increasing concentrations of Hg and Se in the liver (**a**) and in the brain (**b**) indicate a bioaccumulation of Hg and subsequently of Se, most probably as a result of the formation of Se-Hg non-bioavailable compounds in both tissues. Calculated molar ratio between Se and Hg (**c**) in the liver (blue circles) and brain (red squares) is above 1 in all investigated animals. The molar ratio of 1 is indicated by red and blue dashed lines in brain and liver, respectively. The difference between molar concentration of Se and Hg appears to reach a constant value reflecting the essential levels of Se concentration. The blue dashed line indicates average Se levels of of 23.4 ± 8.6 μmol kg^−1^, excluding data from whale 17, which was identified by Grubb’s test as an outlier. The analysis of total Hg was performed by CV-AFS and total Se was determined by ICP-MS.

MeHg binds strongly to biothiols and bioselenols and its excretion rate from the organism is very slow^18^. In general, the dietary intake of MeHg outweighs the excretion capabilities and its bioaccumulation can be observed. To determine the extent of Hg bioaccumulation in organs of stranded pilot whales, we conducted total Hg analysis in liver (n = 21) and brain (n = 11) of 21 members of the stranded pod, which covered a lifespan of 1–36 years. We found significant bioaccumulation of Hg with age in the liver (Fig. 1a) and in the brain (Fig. 1b), with R^2^ = 0.972 and 0.946 respectively.

The essential Se would normally undergo homeostatic regulation and thus should not show bioaccumulative tendencies, however it is retained in the organism in the form of non-bioavailable compounds. Therefore, a positive correlation between age and total Se in the liver (Fig. 1a; R^2^ = 0.955) and brain (Fig. 1b; R^2^ = 0.838) was observed. Interestingly, while the accumulation of Hg in the liver closely mirrors that of Se from the early age (Fig. 1a), higher Se concentrations are found in the brain up to early adulthood (ca. 25–30 years, Fig. 1b). Based on this observation, we hypothesize that selective production of Se-proteins may take place in the brain to maintain the essential levels of Se-proteins, which play a vital role in the brain development of young individuals.

The Se:Hg molar ratio was found to be greater than 1 in all studied animals, with a significantly higher molar Se concentration compared to Hg in the younger individuals, ranging from ~1.5 to >8 for liver and ~6 to >40 for brain. In the adult whales, Se:Hg molar ratios approached 1 and a significant negative correlation with age was observed (Fig. 1c, Supplementary Tables S1 and S2). Interestingly, the difference between the molar concentration of Se and Hg in the brains appears to reach a constant value, of 23.4 ± 8.6 μmol kg^−1^ (Fig. 1d), which could be indicative of an essential levels of Se in the brain.

### Impact of MeHg detoxification on Se-proteins

**Figure 2:**
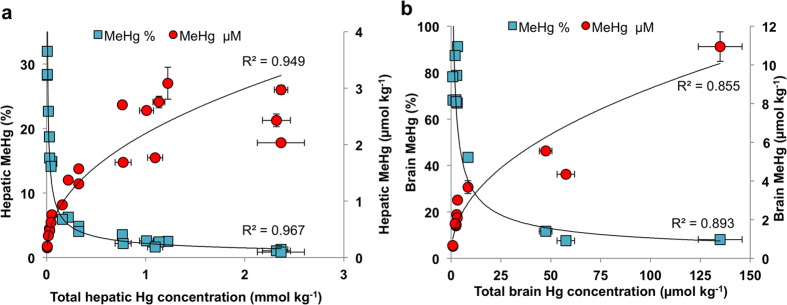
MeHg is demethylated in the liver and brain of pilot whales. Increase of absolute MeHg concentration positively corresponds with total Hg concentration, as a proxy for the age of whales and directly reflects the faster dietary influx of MeHg than natural processing from the liver (**a**) and brain (**b**). The gradually moderate slope of MeHg accumulation in older age suggests slower rate of accumulation thus higher efficiency of the demethylation mechanism in the adult whales. Local demethylation of MeHg in the liver (**a**) and in the brain (**b**) is clearly demonstrated by decreasing percentage of methylated fraction of total Hg, advocating similarities in the response to high MeHg stress. MeHg analysis was performed by species-specific isotope dilution GC-ICP-MS.

MeHg present in the gastrointestinal tract is transported across the luminal plasma membrane of enterocytes into the bloodstream, which delivers a large portion of MeHg bound to erythrocytes to the liver, where its demethylation takes place^19^,^20^. However, a fraction of MeHg, which is not sequestered by hepatocytes continues to circulate in the blood stream and can be transported across the blood-brain barrier^21^. It is thought that once MeHg is within the brain, detoxification takes place locally. We therefore examined the extent of MeHg detoxification in the liver and brain tissues, which can provide us with the tolerable levels of MeHg and the kinetics of the detoxification process. Our analysis showed that the absolute concentration of MeHg is positively correlated with the total Hg concentration in both liver and brain tissues (Fig. 2a,b). By contrast, the percentage of the methylated fraction of total Hg showed an inverse relationship with the age of the organism (Fig. 2a,b), meaning that the percentage fraction of MeHg is decreasing with the increase of total Hg. Interestingly, a portion of the total Hg in the brain, which was not susceptible to derivatisation also demonstrated an age-related accumulation (Supplementary Fig. S1), suggesting that Hg formed an inert compound most probably as a result of MeHg degradation. Moreover, we found significant concentrations of inorganic Hg in the brain tissue (Supplementary Table S1), possibly an intermediate product of demethylation reactions, as inorganic Hg cannot cross the blood-brain barrier^21^,^22^,^23^.

Se-cysteine is biosynthesised in the cytoplasm from inorganic Se, whose levels are maintained by Se-methionine, the biological pool of Se^13^. The biosynthesis proceeds through the formation of several intermediate Se-biomolecules, including Se-cystathionine. Following the suggestions that detoxification of MeHg results in formation of Hg and Se containing compounds, we investigated the influence of MeHg on the levels of various Se-species by using high performance liquid chromatography hyphenated with inductively coupled plasma mass spectrometry in tandem with electrospray ionisation mass spectrometry (HPLC-ICP-MS/ESI-MS), simultaneously for elemental and molecular Se. Although we were unable to identify all of the compounds (Supplementary Figs S2 and S3), the results showed that the organisms maintained similar Se-cysteine concentration in the juvenile and adult liver and brain (Table 1). In contrast, Se-methionine was depleted below the instrumental limit of detection (<5.5 ng g^−1^) in the adult animals, whereas the concentration of Se-cystathionine and inorganic Se (Se-DTT) were significantly increased in comparison with the juvenile tissue.

### Characterisation of natural HgSe nanoparticle and Se-Hg clusters

**Figure 3:**
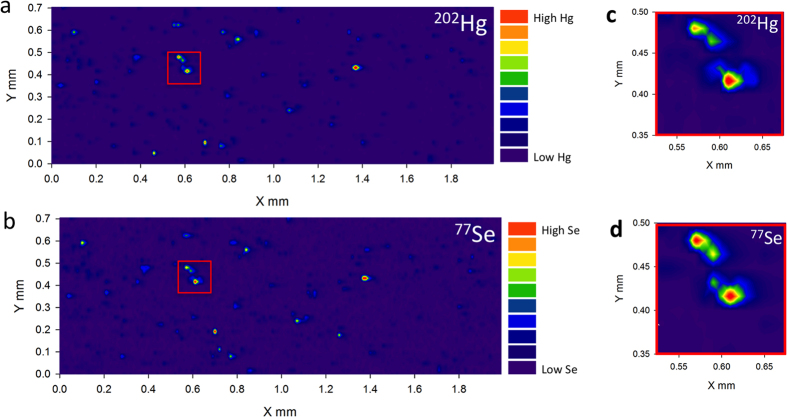
*In vivo* formation of HgSe nanoparticles. 2D map of Hg (**a**) and Se (**b**) distribution in the brain tissue of an adult whale generated by LA-ICP-MS. Co-localisation of Hg and Se hotspots suggests formation of particles containing both elements (**c**,**d**). The legend indicates the intensity of monitored *m/z* in counts per second (cps) with the lowest intensity represented by blue colour and the highest intensity in red colour.

The proposed mechanism of MeHg detoxification most probably starts by exchange of the binding ligand (MeHg-S-R) and formation of MeHg-Se-R compounds, ultimately leading to deposition of inert HgSe and formation of larger clusters or crystals containing both elements^20^. To validate this hypothesis, we used laser ablation ICP-MS (LA-ICP-MS) mapping to examine the distribution of Hg and Se. Bio-images of the ablated brain and liver thin sections showed matching elemental patterns for Hg and Se, confirming the co-localization of these elements (Fig. 3a–d and Supplementary Fig. S4). Furthermore, the size variation of the hot spots observed on the images indicates formation of clusters of a wide size range containing both elements, Hg and Se.

**Figure 4:**
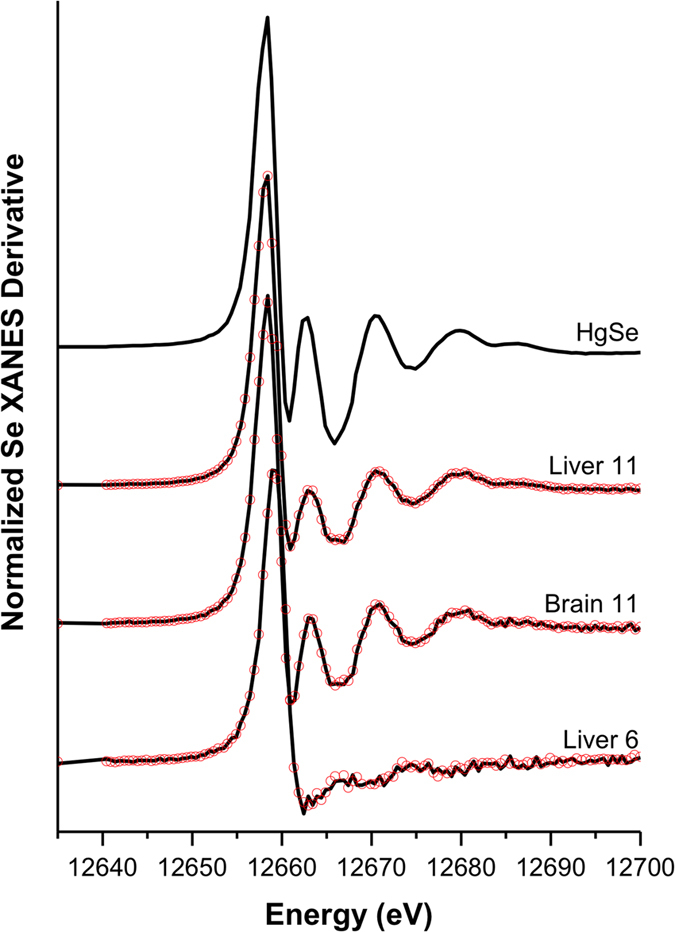
Identification of bioformed HgSe nanoparticles. Synchrotron-based XANES analyses show high similarities between Se signal generated by a HgSe standard and Se signal from the liver and brain of an adult whale (Liver 11, Brain 11), indicating high proportions of Se being locked in HgSe aggregates. Absence of HgSe aggregates was found in the juvenile liver (Liver 6) as Se signal doesn’t show similarities with HgSe standard. Black solid lines show the experimental data and red dots are the best fit of the linear combination fitting.

To corroborate the identity of Se-Hg clusters, we used synchrotron-based X-ray absorption near edge structure (XANES) spectroscopy. Linear combination fitting of Hg L_III_- spectra showed that most of the Hg exists as Hg(II) and considering the similarity of the XANES spectra, the local structures around the Hg atoms seem to be similar to those in HgSe in both the brain and liver of adult whales (Supplementary Fig. S5, Supplementary Table S3). However, Hg concentration in the youngest individuals was not high enough to obtain good spectra. This result was additionally confirmed by the Se K-edge spectra (Fig. 4, Supplementary Table S3) as the shape and chemical shifts indicate that Se exists as Se(-II), and they resemble the HgSe spectrum with the exception of the sample from the youngest individual.

**Figure 5:**
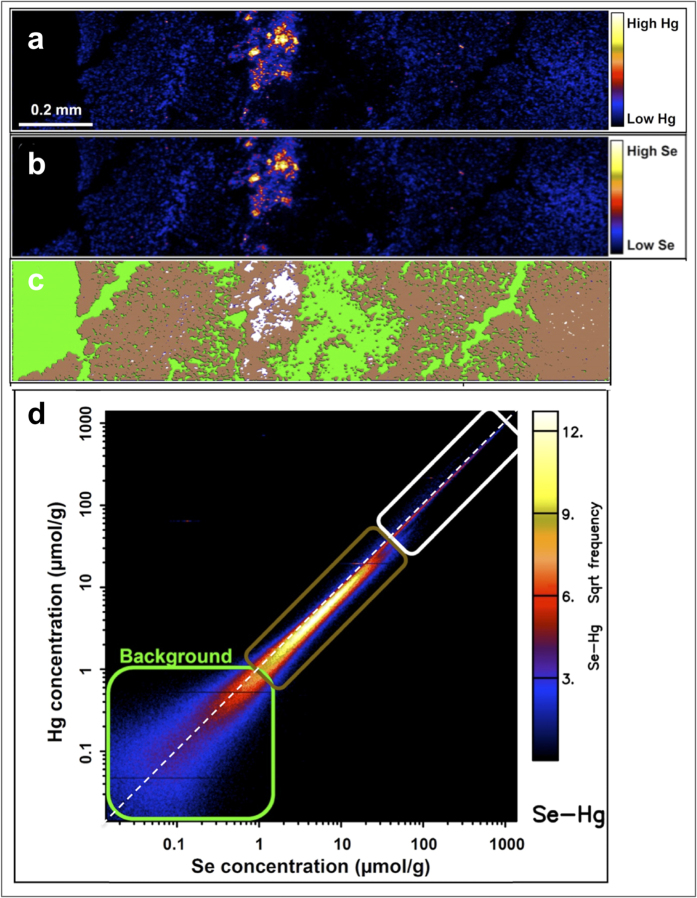
Interaction of Hg and Se of Se-Hg clusters. 2D map of Hg (**a**) and Se (**b**) distribution in the liver of an adult whale generated by synchrotron μ-XRF from a 30 μm thin section. Hg clusters are visible in the areas of high Hg intensity (yellow/white), which corresponds with Se distribution. Diffused Hg and Se is illustrated in blue. In **c** the elemental map of Hg from (**a**) is overlaid with the three pixel populations identified using the ‘elemental association’ module in GeoPIXE, with each pixel population corresponding to the coloured boxes in the element correlation plot in (**d**). Se:Hg molar ratio of 1 is highlighted in (**d)** by a diagonal white dashed line. The white rectangle is corresponding to white pixels in the map (**c**), the aggregates in the map (**a**) with Se:Hg molar ratio of 1. Areas of low Hg concentration (blue in map (**a**), represented in brown in map (**c**) show skewed elemental association towards higher Se in the plot (**d**) with Se:Hg molar ratio >1.

As a further demonstration of the presence of Se-Hg clusters, the tissue was analysed using highly sensitive synchrotron-based μ-X-ray fluorescence microscopy (μ-XRF). The image of a juvenile liver showed diffused Hg and Se with Se-Hg clusters being absent, what could be a result of insufficient lateral resolution (Supplementary Fig. S6). In contrast, the adult liver revealed distinct aggregation of Se-Hg particles forming clusters of up to 5 μm in size (Fig. 5a,b). The elemental association plot (Fig. 5d) shows strong correlation between Hg and Se in the mapped tissue, but interestingly, the highest frequency of Se-Hg occurrence was found in the areas surrounding the large clusters rather than in the clusters themselves (Fig. 5c,d, brown colour). Furthermore, calculated Se:Hg molar ratios in the area surrounding the clusters is skewed towards higher Se concentration, deviating from the molar ratio of 1, but interestingly, the molar ratio in the actual clusters (Fig. 5c,d, white colour) decreased to 1.

**Figure 6:**
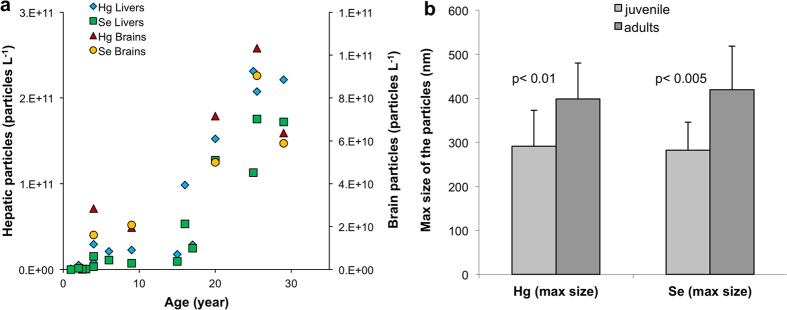
Number and size of Se and Hg containing particles in the liver and brain is increasing during the lifespan of pilot whales. Increasing number of particles with increasing age (**a**) indicates positive demethylation and deposition of unreactive Hg in the form of Se-Hg aggregates as a result of detoxification. (**b**) Shows the paired t-test between the maximum size of Hg and Se particles in the juvenile and adult liver tissue. Significant difference between the particle sizes advocate aggregation of the particles and formation of large clusters over the lifetime of the animals. The data were collected by ICP-MS in the single particle mode.

Given that the pixel size of the μ-XRF image was 800 nm, the size distribution of nano-scale Se-Hg clusters was investigated by single particle ICP-MS (spICP-MS). As simultaneous detection of Hg and Se in the individual particles was not possible, the particle size distribution was based on separate measurements, monitoring either Se or Hg. However, despite this technical limitation, the analysis showed that there is an increasing number of particles correlating with the age of the individual animals (Fig. 6a, Supplementary Table S4). Additionally, statistical comparison between the maximum size of the particles in the juvenile and adult whale tissues showed a significant difference (Fig. 6b, Supplementary Table S4). Hence, the particles get larger with age.

## Discussion

Strong binding affinity between Hg and Se^24^ reduces the bioavailability of Hg and potentially mitigates its toxicity, but consequently, may also inflict Se deficiency. It has been widely reported that a molar ratio between Se and Hg greater than 1 implies that Se is providing protection against Hg toxicity, while a ratio below 1 reflects very limited detoxification ability of Se^25^,^26^,^27^. Considering that the biochemistry of MeHg detoxification is largely unknown, the use of Se:Hg molar ratio to identify the detoxification abilities of an organism may prove to be flawed. Such an assumption would hold if the only Hg species present in the tissue would be in the form of HgSe particles, and thereby it would be plausible to assume that organism with molar ratio above 1 has enough bioavailable Se to support vital biological functions. However, if Hg binds to more than 1 Se atom during the bioformation of HgSe particles, this assumption would result in overestimation of the protection provided by Se. Our study found that the Se:Hg molar ratio in small particles is greater than 1 and only after the formation of large clusters, the ratio is reduced to 1 (Fig. 5d). This observation could be explained by binding of HgSe particles to Se-rich proteins. Presumably, Se-protein P, which is one of the major selenoproteins found in the plasma, may form the backbone of these aggregates^28^. Under such circumstances, the biological functions of Se-protein P may be hindered by the formation of HgSe particles and it is unknown whether such impediment would promote increased expression of Se-protein P to counteract its reduced bioavailability.

The relatively low percentage of the methylated fraction of total Hg found in the tissues of juvenile whales suggests (Supplementary Tables S1 and S2), that the detoxification process is fully developed from the early age of the animals, which is in contradiction with previous suggestions that young animals are not able to efficiently demethylate MeHg^29^. Additionally, several authors^30^,^31^ suggested that initiation of a demethylation process requires the total Hg concentration to exceed a certain threshold value. In striped dolphin, the threshold value was suggested to be 0.49 μmol of total Hg kg^−1^ of wet weight of liver^32^. However, our data indicate that the full cycle of Hg and Se particles bioformation is completed in the early age of the animals with total hepatic Hg concentrations as low as 0.027 μmol kg^−1^ of wet weight, as Hg and Se containing particles were found in the juvenile tissues (Supplementary Tables S1 and S2). Therefore, the lack of observed demethylation products in tissues with total Hg concentration below the suggested threshold values could be explained by kinetic rates of the reactions. Transport of MeHg to the bloodstream provides the means of circulation within the biological system, but, as the excretion through urine is very limited, demethylation must take place in organs such as liver and brain. In general the rate of MeHg detoxification is dependent on the distribution kinetics of dietary MeHg between individual organs, which was found to be surprisingly slow^33^ and rate of actual demethylation reactions. The latter leads to the strong binding of MeHg to bio-thiols, particularly glutathione^34^ but ultimately must result in the formation of Hg-Se-cysteine compounds. Considering the biological importance of Se-cysteine, its abundance in the living organism and complexity of the metabolic pathway, it can be assumed that the kinetic rate of these reactions is very slow.

A strong nucleophilic property of Se-cysteine makes it the natural target for binding by electrophilic Hg and therefore, it could be expected that the onset of MeHg toxicity will be reflected by a decrease in Se-cysteine concentrations. The comparison between Se-cysteine levels in the adult and juvenile liver and brain tissue showed very similar concentrations despite the significantly different Hg levels and degree of detoxification (Tables 1 and S3). Similarly, the molar difference between total Se and Hg in the brain resulted in the constant Se concentration along the studied pod (Fig. 1d), also identified by other authors^35^. These observations suggest that there are essential levels of Se-cysteine necessary to support vital biological functions, which require its continuous supply. In contrast to other amino acids, Se-cysteine is not recycled but degraded to inorganic Se, which is used for the *de-novo* synthesis^13^. Healthy organisms retain Se reserves in the form of Se-methionine, which tend to mitigate the harmful effects caused by MeHg exposure by maintaining the supply of Se lost to MeHg binding^36^. However, if the rate of MeHg uptake is constantly higher than uptake of Se, this reserve may become depleted. Therefore, it is conceivable that MeHg toxicity is firstly reflected in increased levels of Se-cysteine intermediate species, followed by depletion of Se-methionine and lastly by a decrease in Se-cysteine concentration. The results in Table 1 show that Se-methionine in the adult brain and liver tissue is depleted below the instrumental limit of detection, likely a result of increased production of Se-cysteine via the Se-cystathionine intermediate, the concentration of which is significantly increased as a direct response to high dietary intake of MeHg.

Several authors^37^,^38^ have suggested that biologically formed Hg and Se containing particles are inert and thus deemed to be non-toxic. XANES analysis of the adult whale tissues confirmed formation of HgSe nanoparticles (Fig. 4), which are possibly bound to Se-rich proteins as suggested by Se:Hg molar ratio above 1. It is evident from μ-XRF mapping (Fig. 5a,b) that these small particles undergo cluster formation, resulting in the reduction of the Se:Hg molar ratio to 1. Such observation could be explained by non-stoichiometric binding of Hg to already formed HgSe nanoparticles attached to proteins. Additionally, several studies suggest incorporation of sulfur in the HgSe nanoparticulate structures, thus mixed chalcogenides are forming^39^,^40^. Although, HgSe is thought to be non-reactive, its nanoparticulate form suggests otherwise. High surface-area-to-volume ratio and low co-ordination number, which increase surface energy of nanoparticles significantly alter the reactivity and behaviour in comparison with bulk material^41^. Hence it is possible that biologically formed HgSe nanoparticles act as nucleation centres for the formation of large Se-Hg clusters, growing during the lifespan of the animals (Fig. 6).

Moreover, as the detoxification mechanism results in the formation of nanoparticles, the actual efficacy of this process could be questioned. There is strong evidence that engineered nanoparticles are able to transverse the cell boundaries and cause organ malfunction^42^. They can be extremely efficient in production of reactive oxygen species and free radicals^43^, which are causing oxidative damage and inflammation and may consequently result in damage of proteins, cell membranes and DNA^44^,^45^. Thus, it is plausible that the formation of HgSe nanoparticles is only a short-term solution to high dietary MeHg uptake and the gaps in the current understanding of the toxicological response to naturally formed nanoparticles should be addressed.

## Conclusion

One of the principal problems in the field of Hg biochemistry is the poor understanding of the Hg mediated toxicological impact on the living organism. Only recently we have come to understand that the role of Se-proteins in the detoxification process is far more complex than mitigation of MeHg’s toxicity via its transformation to less toxic species. Our data show that accumulation of MeHg in the brain of juvenile whales may promote preferential supply of Se to the brain at the expense of other tissues, in order to maintain bioavailability of essential Se during the development stage. The evidence of similar concentration levels of Se-cysteine in the liver and brain, which seem to be independent of the animals’ age, is particularly interesting when compared with the increasing concentration levels of MeHg in both tissues. It appears that long-term dietary exposure to MeHg has a direct impact on the biosynthesis of Se-proteins, particularly depletion of Se-methionine in the adult whales as a consequence of Se-cysteine sequestration. We also found that the detoxification process is fully developed in young animals and takes place in both the livers and brains via the metabolism of nanoparticulate HgSe formation, although we are not able to say whether it proceeds through identical pathways. It appears that HgSe nanoparticles are bound to Se-rich structures, which are core centres for the formation of Se-Hg clusters. However, the process of HgSe cluster formation in organs is not yet fully understood, it is likely that high reactivity of HgSe nanoparticles promotes binding of Hg to their surface, resulting in the observed growth of the clusters over the lifetime of the animal.

## Materials and Methods

### Sample collection

A pod of long-finned pilot whales stranded on the beach between Anstruther and Pittenween in Scotland, United Kingdom on 12^th^ September 2012. The dead pilot whales (16 females and 5 males) were part of a much larger pod of which 31 animals stranded and 10 were successfully refloated. It was therefore not possible to identify mother-calf pairs. The whales were autopsied at the stranding site shortly after death. The length of the animals was recorded and teeth were extracted for ageing. The age was determined from extracted teeth (16 of the 21 individuals) according to method described elsewhere^7^. Tissues of liver, kidney, muscle, blubber and brain frontal lobe were sampled and stored at −8 °C. Prior to analysis samples were freeze-dried and homogenized.

#### Determination of total Se concentration

Freeze-dried and homogenised tissue (~0.1 g) was digested in HNO_3_ (5 mL, 68%) in pressurised closed vessels (Mars 5, CEM Corporation, UK). The microwave programme was as follows: step 1, room temperature to 200 °C, 20 min; step 2, 200 °C, 30 min. After the completion of the microwave programme, the digests were allowed to cool to room temperature and were stored at 4 °C until elemental analysis. Sample analysis was performed by ELEMENT 2 ICP-MS (Thermo Fisher Scientific, Germany). Prior to measurement, the digests were diluted in bi-distilled water to a final acid concentration of 5% and m/z ^77^Se, ^82^Se and ^74^Ge were monitored during the measurement in high-resolution mode.

#### Determination of total Hg concentration

Cold vapour atomic fluorescence spectroscopy (PS Analytical Ltd, UK) was used for Hg analysis. The digested samples (as described in the previous section) were diluted to 5% acid concentration prior to measurement. The diluted digest was first mixed with the blank solution (5% HNO_3_), followed by reaction with reductant (2% SnCl_2_) in the sample valve. This reaction resulted in vapour generation of elemental Hg, which was purged with argon gas from the gas-liquid separator through the dryer into the atomizer.

#### Laser Ablation Inductively Coupled Plasma Mass Spectrometry (LA-ICP-MS)

LA-ICP-MS is an ideal technique for two-dimensional mapping of trace elements in the tissue. Therefore, it was used to generate a map of the spatial distribution of Hg and Se within liver and brain tissue. A New Wave UP-213 system operating at 213 nm wavelength was used for ablation of 15 μm thin section produced using a cryo-mictrotome (Bright 5030 Microtome). The tissue was placed in the SuperCell^®^ and ablated in the scan mode using a 5 μm spot size with scan speed of 25 μm s^−1^, 20 Hz frequency and 45% energy with resulting fluence of 0.7–1.1 J cm^−2^. The ablated particles were transported by a constant stream of argon gas directly to a 7500c ICP-MS system (Agilent, Japan). Mass-to-charge ratios of ^202^Hg, ^77^Se and ^78^Se were monitored. The images were generated using SigmaPlot version 11 and all data was corrected for background values.

#### X-ray fluorescence elemental mapping

Thin sections (30 μm thick) were placed between two layers of 4μm thick Ultralene film and analysed at the X-ray Fluorescence Microscopy (XFM) beamline at the Australian Synchrotron^46^. This undulator beamline is equipped with a Si (111) monochromator and Kirkpatrick-Baez mirrors focusing the beam to a spot size of approximately 2 μm^2^. Elemental maps were collected at 18.5 keV using a 384-element Maia detector in a backscatter geometry^47^. The samples were analysed continuously in the horizontal direction (‘on the fly’) with steps of 0.8 μm in the vertical direction. The sample stage was set to a speed of 0.064 to 0.15 mm s^−1^ resulting in a pixel (0.8 μm) transit time of approximately 5–13 ms. The full XRF spectra were then analysed using GeoPIXE^48^,^49^. This software uses Dynamic Analysis to subtract background and resolve overlapping peaks when generating elemental maps, thus allowing calculation of semi-quantitative values for all the different elements. Various correlation regions for Hg and Se on the maps were identified using the ‘Element Association’ function of GeoPIXE.

#### Species-Specific Isotope Dilution Gas Chromatography Inductively Coupled Plasma Mass Spectrometry (SSID-GC-ICP-MS)

To assess the accumulation of MeHg and potential demethylation of this species Hg speciation was performed as described elsewhere^50^. Briefly, tissue (~50 mg) was solubilized in TMAH (5 mL, 25% w/w) using an accelerated reaction system microwave (Mars 5, CEM Corporation, UK). One mL of the solubilized tissue was spiked with ^201^MeHg^+^ and ^199^Hg^2+^ and was allowed to equilibrate for 30 min, then buffered with 5 mL of 0.1 M acetate buffer to set the pH to 3.9. One mL of iso-octane was added to the mixture followed by 1 mL of 1% (m/v) solution of NaBPr_4_. Vials were centrifuged for 10 min at 3,747 × g and the organic layer was transferred into GC amber vial and stored at −20 °C until analysis. Analysis was performed using HP-6890 gas chromatogram (Agilent Technologies, USA) hyphenated to 7500c ICP-MS (Agilent Technologies, USA) via a heated silcosteel^®^ transfer line (Thames Restec, UK). The intensities of *m/z*^199^Hg, ^200^Hg, ^201^Hg, ^202^Hg, ^203^Tl, and ^205^Tl were monitored in the transient signal mode and all integrated Hg peak areas were mass bias corrected using *m/z*^203^Tl, and ^205^Tl following the method described by Russell *et al*.^51^.

#### High Performance Liquid Chromatography Inductively Coupled Plasma Mass Spectrometry (HPLC-ICP-MS) and Electrospray ionization mass spectrometry (ESI-Orbitrap-MS)

In order to evaluate the effect of Hg accumulation on the Se species the tissue was subjected to enzymatic digestion and Se species were derivatised for subsequent analysis by HPLC-ICP-MS. The detailed analytical procedure is described below. Freeze-dried tissue (~100 mg) was pre-treated with 5 mL of binary solvent (MeOH/DCM in 1:2 ratio) and sonicated for 5 min. Samples were then centrifuged, the supernatant decanted and any residual solvent in the tissue evaporated. Subsequently, ammonium acetate buffer (2 mL, 10 mM, pH 7) was added to the tissue, followed by the addition of dithiothreitol (200 μL, 100 mM), iodoacetamide (400 μL, 100 mM) and incubated in the dark for 5 min. Afterwards, 2 mL of protease enzymes (10mg mL^−1^) was added and the mixture was incubated at 37 °C for 24 hours. The hydrolisates were then centrifuged at 12,513 × g for 15 min and the supernatant was collected and stored at 4 °C for the HPLC analysis. HPLC 1100 series (Agilent Technologies, USA) was used for Se species using an Agilent Zobax Eclipse XDB C-18 (150 × 4.6 mm id, 5 μm) column. Separation was achieved using a gradient elution with eluent A 0.1% formic acid in water and eluent B 0.1% formic acid in methanol. The gradient programme went as follows: 0–8 min 100% A, 8–15 min 100% B, 15–22 min 100% A. The eluate from the column was split, with 25% introduced directly to the 8800 ICP-MS (Agilent Technologies, Japan) and 75% going to the LTQ-LX Orbitrap Discovery (Thermo Fisher Scientific, Germany). This system provided us with an accurate mass of the Se in the sample, in addition to species separation and subsequent identification.

#### X-ray absorption near edge structure (XANES) spectroscopy

XANES spectroscopy measurement was conducted at the Materials Research Collaborative Access Team (MRCAT) beamline Sector 10-ID, at the Advanced Photon Source of Argonne National Laboratory (USA) for Hg and Se speciation. The beam size was 750 × 750 μm and the samples were solids from freeze dried tissues. The storage ring operated at 7 GeV in top-up mode. A liquid N_2_ cooled double crystal Si (111) monochromator was used to select the incident photon energies and a platinum-coated mirror was used for harmonic rejection. Calibration was performed by assigning the first derivative inflection point of L_III_ edge of gold foil (11919 eV) for Hg scans with periodic recalibration to ensure stability, and of the K edge of elemental Se (12658 eV) for Se scans with simultaneous collection for each scan for calibration of spectra. The elemental Se utilized was a crystalline hexagonal structure with a metallic gray color. Five Hg L_III_ (12284 eV) or Se K (12658 eV) edge X-ray absorption spectroscopy spectra were collected using a 4-element Vortex florescence detector. Data analysis was conducted using Athena software^52^. Replicate scans for each sample were merged and then normalized. Linear combination fitting (LCF) was used for the identification of Hg and Se species in the samples. Linear combination fits (−30 to + 70 eV relative to the calibration energy) were performed using XANES derivative μ(E) spectra from reference standards to Hg and Se phases in the tissue samples. During the LCF, components were only allowed to contribute to the model if the sum-square error was reduced by 20%^53^. The R-factor is a measure of the mean square sum of misfit at each data point and describes the degree of uncertainty in the fitting process^54^. Most standards were solids, measured in both fluorescence and transmission mode. The concentration of the standards was calculated to permit one absorption unit, blended with a binding agent, and pressed into a pellet for analysis. Reference materials for Hg LCF included mercury selenide (HgSe), cinnabar (α-HgS), metacinnabar (β-HgS), mercurous sulphate (Hg_2_SO_4_), mercuric sulphate (HgSO_4_), mercurous oxide (Hg_2_O), and mercuric oxide (HgO), while methylmercury (CH_3_HgCl,), glutathione-methylmercury (MeHg-GS) and glutathione-mercuric chloride (GS-Hg-Cl) were measured in solution. Selenium LCF reference standards included mercury selenide (HgSe), selenocystine, selenocysteine^55^, selenomethionine, methylselenomethionine, trimethylselenonium, γ-glutamyl-Se-methylselenocysteine, sodium selenate, and sodium selenite. Data for LCF fits reveal Hg and Se speciation in each sample as ratios of these mineral forms.

#### Single Particle Inductively Coupled Plasma Mass Spectrometry (sp-ICP-MS)

To liberate particles from the matrix, freeze-dried tissue was subjected to enzymatic digestion. Freeze-dried tissue (~20 mg) was firstly defatted, and then the enzyme solution (protease was dissolved in 50 mM ammonium bicarbonate buffer (pH 7.4) to a final concentration of 1 mg mL^−1^ with 5 mg mL^−1^ of SDS) was added. Digestion solution was mixed with the tissue and incubated at 37 °C overnight. In order to separate Se-Hg particles from dissolved Hg and Se, the digests were subjected to a cleaning process as follows. Firstly, the samples were passed through a centrifugal filter (Amicon Ultra) with 50 kDa cut-off by spinning at 12,513 × g for 3 min. The retained fraction of approximately 0.1 mL was then diluted with MilliQ water to 0.5 mL and spun again in order to wash out any dissolved Hg and Se. The concentration of Hg in the flow-through was monitored by CV-AFS and the cleaning process was repeated until no Hg was detected. For particle size determination, a model 7900 ICP-MS (Agilent Technologies, USA) was operated in the single particle mode (3 ms dwell time) according to a method described elsewhere^56^. The transport efficiency was determined by measuring NIST AuNP suspension with a known average particle diameter and particle number concentration. Digests were diluted prior to measurement in MilliQ water. Following the measurement of each sample, MilliQ water was analysed to monitor any possible carry over from the previous measurement. Only Hg and Se were measured for single particle analysis in separate analyses, from the same tissue homogenate.

## Additional Information

**How to cite this article**: Gajdosechova, Z. *et al. In vivo* formation of natural HgSe nanoparticles in the liver and brain of pilot whales. *Sci. Rep.* **6**, 34361; doi: 10.1038/srep34361 (2016).

## Supplementary Material

Supplementary Information

## Figures and Tables

**Table 1:** List and concentrations (μg kg^−1^) of identified Se species in the selected tissues of pilot whale samples analysed by HPLC-ICP-MS/ESI-MS.

Sample ID	[Se-Cys]_2_	Se-Cys-CAM	Se-DTT	Se-Met	S-Me-SeCys	Se-cystathionine
Brain 6	21.4	426	203	69.7	110	21.0
Brain 11	96.0	430	1225	LOD	118	47.1
Liver 6	156	554	295	78.8	117	42.2
Liver 11	LOD	574	16651	LOD	385	1516

Analysis was carried out on two whales, representing the age extremes, juvenile whale 6 (ca. 1 year old) and adult whale 11 (28 years old). Similar levels of Se-Cys-CAM in both whales, independent of age and Hg concentration indicate biologically relevant levels of Se-Cys necessary for survival of the animal. Faster rate of Se-Cys production in the adult whale is reflected in the significantly higher concentration of Se-cystathionine and H_2_Se (as Se-DTT), which has a direct effect on the depletion of Se-Met. Exceptionally high levels of Se-DTT in the adult tissue may also mirror the presence of Se-Hg clusters. It is possible that some of the Se present in the particles was reduced during the derivatisation and formed Se-DTT. LOD means instrumental limit of detection, which was (<5.5 ng/g). The following compounds were identified by ESI/MS: [Se-Cys]_2_ (selenocystine), Se-Cys-CAM (carbamidomethylated selenocysteine), Se-DTT (seleno-dithiothreitol), Se-Met (selenomethionine), S-Me-Cys (S-methyl-selenocysteine), Se-cystathionine (selenol-cystathionine).
