# Supplementary material for: In vivo formation of natural HgSe nanoparticles in the liver and brain of pilot whales
Source: Sci Rep. 2016 Sep 28;6:34361. doi: 10.1038/srep34361 (PMC5039623; doi:10.1038/srep34361)
Supplement: Supplementary Information [file srep34361-s1.pdf]

Supplementary Information for

In vivo formation of natural nanoparticles in the liver and brain of pilot whales

Zuzana Gajdosechova[a], Mohammed M. Lawan[a], Dagmar S. Urgast[a], Andrea Raab[a], Kirk G. Scheckel[b], Enzo Lombi[c], Peter M. Kopittke[d], Katrin Loeschner[e], Erik H. Larsen[e], Glenn Woods[f], Andrew Brownlow[g], Fiona L. Read[h], Jörg Feldmann[a], Eva M. Krupp[a]

[a]Trace Element Speciation Laboratory, Department of Chemistry, Meston Walk, University of Aberdeen, Aberdeen, AB24 3UE, UK. [b]United States Environmental Protection Agency, National Risk Management Research Laboratory, 5995 Center Hill Avenue, Cincinnati, OH 45224, USA. [c]Centre for Environmental Risk Assessment and Remediation, University of South Australia, Building X, Mawson Lakes Campus, Mawson Lakes, 5095 South Australia. [d]School Agriculture and Food Sciences, The University of Queensland, St Lucia , Queensland, 4072, Australia. [e]National Food Institute, Technical University of Denmark, Mørkhøj Bygade 19, DK-2860 Søborg, Denmark. [f]Agilent Technologies Ltd, 5500 Lakeside, Cheadle, SK8 3GR, UK. [g]SAC Wildlife Unit, Inverness, UK. [h]Oceanlab, University of Aberdeen, Main Street, Newburgh, Aberdeenshire, AB41 6AA, UK.

*corresponding author: Eva M. Krupp, Email: e.krupp@abdn.ac.uk

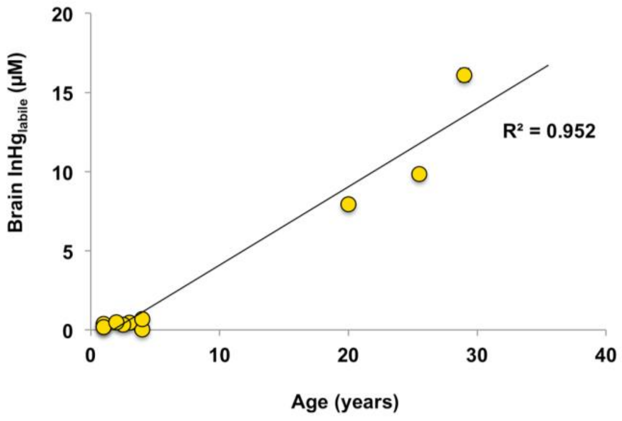

Figure S1. Evidence of MeHg demethylation in the brain of stranded pilot whales. Increasing concentration of labile inorganic Hg in the brain indicates positive demethylation mechanism as inorganic Hg cannot cross the blood brain barrier. Labile inorganic Hg is defined as fraction of inorganic Hg susceptible to derivatisation.

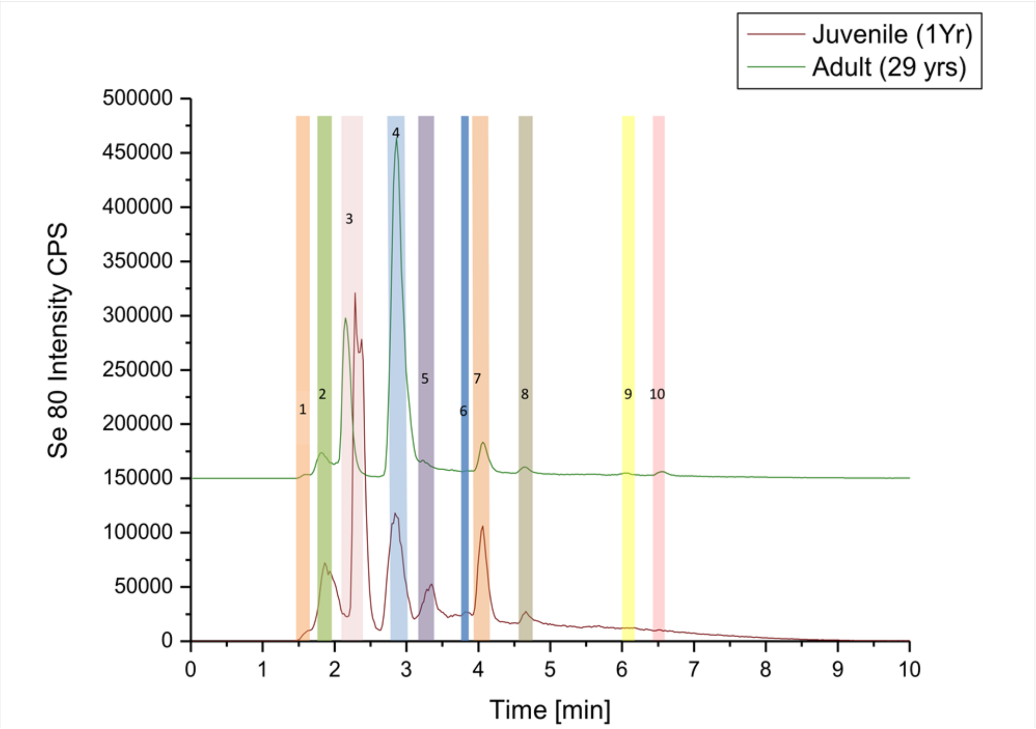

Figure S2. Chromatogram generated by reverse phase HPLC-ICP-MS of whale brain tissues with separated Se species. 1= unk 1, 2= $[SeCys]_2$, 3= SeCys-CAM, 4= Se-DTT, 5= selenomethionine, 6= unk 2, 7= S-methyl-SeCys, 8= Seleno-cystathionine, 9= unk 3, 10= unk 4.

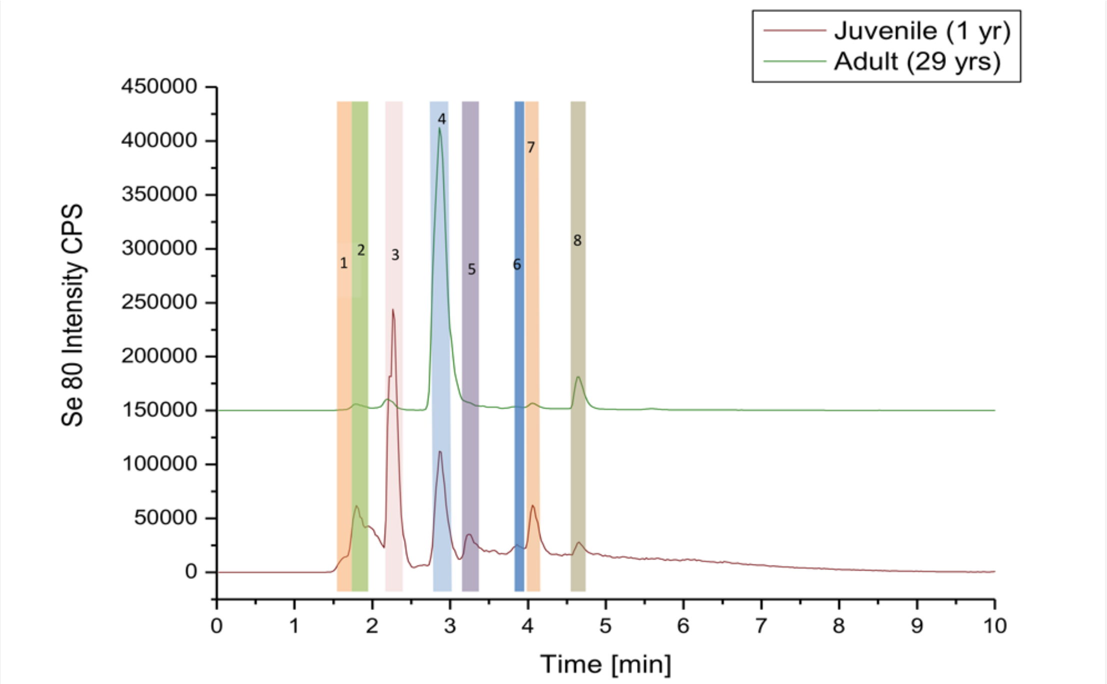

Figure S3. Chromatogram generated by reverse phase HPLC-ICP-MS of whale liver tissues with separated Se species. 1= unk 1, 2= $[SeCys]_2$, 3= SeCys-CAM, 4= Se-DTT, 5= selenomethionine, 6= unk 2, 7= S-Me-SeCys, 8= Seleno-cystathionine.

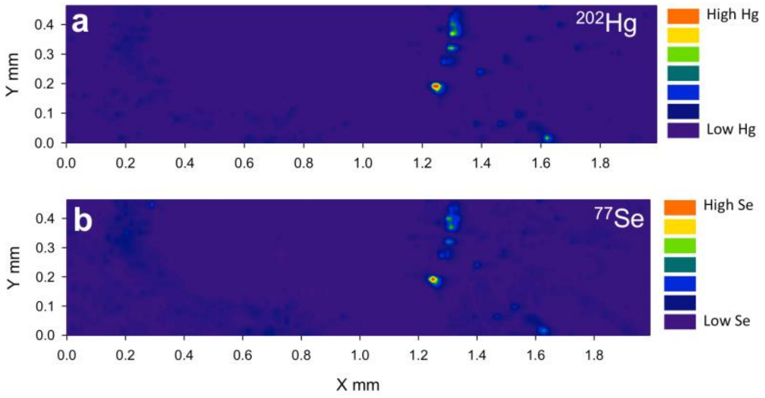

Figure S4. Localisation of particles containing Hg and Se. 2D map of ^{202}Hg **(a)** and ^{77}Se **(b)** distribution in 15 µm thin section of the adult whale liver tissue. Elemental map generated by LA-ICP-MS shows hotspots of high ^{202}Hg and ^{77}Se intensities in yellow/red colour, which indicate aggregation of these elements. Location of the hotspots on the individual maps suggests co-localization of Hg and Se within the same aggregates.

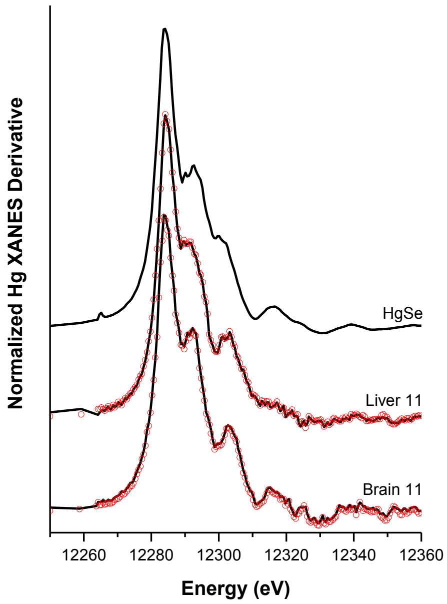

Figure S5. Identification of bioformed HgSe nanoparticles. Synchrotron generated Hg L_{III} XANES spectra of HgSe standard and whale tissues. Experimental data is shown as solid lines and the red points show the linear combination fitting. Strong similarities in the spectra of a HgSe standard and the liver and brain of an adult whale (Liver 11, Brain 11), indicate high proportion of Hg being present in the form of HgSe particles.

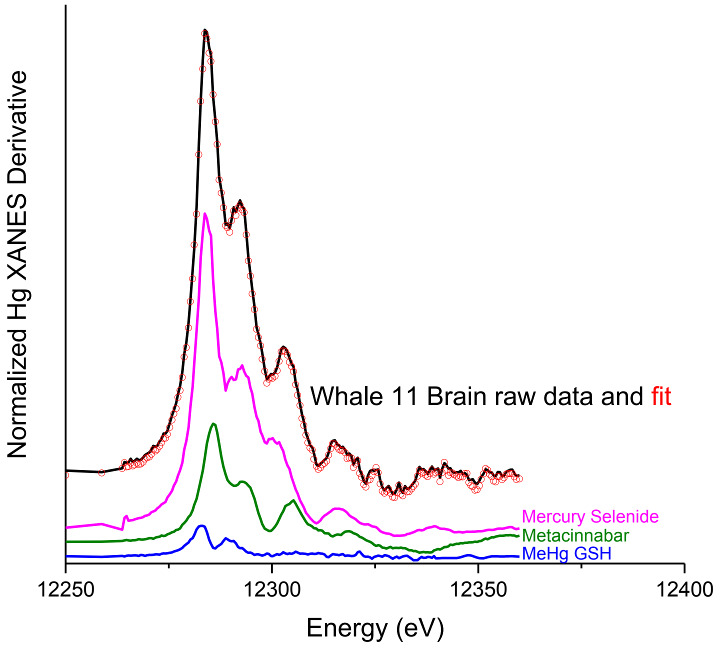

Figure S6. Identification of bioformed HgSe nanoparticles. Synchrotron generated Hg L_{III} XANES spectra of selected Hg standards and adult whale brain tissues. Experimental data is shown as solid lines and the red points show the linear combination fitting.

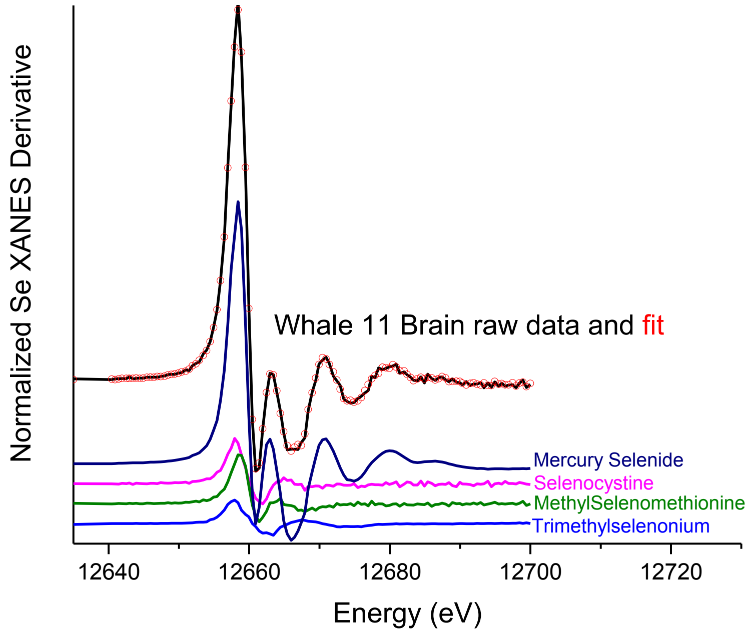

Figure S7. Identification of bioformed HgSe nanoparticles. Synchrotron generated Se K-edge XANES spectra of selected Se standards and adult whale liver tissues. Experimental data is shown as solid lines and the red points show the linear combination fitting.

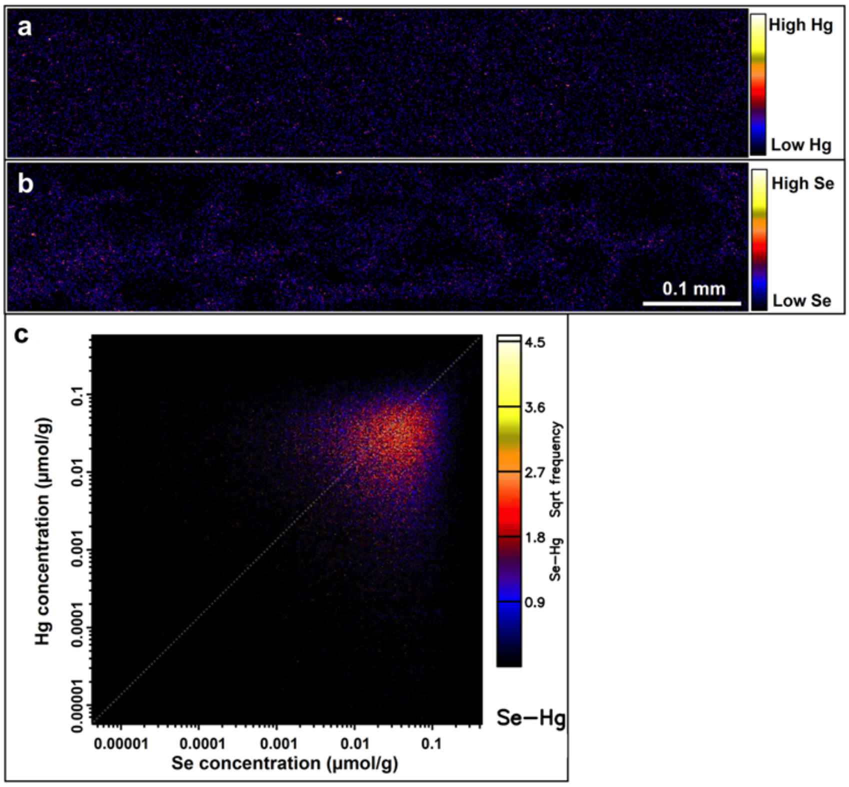

Figure S8. Interaction of Hg and Se of Se-Hg clusters. a Hg distribution map in 30 µm thin section of juvenile whale liver generated by synchrotron µ-XRF shows absence of Hg aggregates. Similar pattern can be seen for Se distribution in panel **b** suggesting slow reaction rate of demethylation mechanism leading to the formation of Se-Hg aggregates in the juvenile whale. Elemental association plot in panel **c** indicates no correlation between Hg and Se. White line indicates Hg and Se molar ratio of 1.

Table S1 Concentrations of measured total Hg, MeHg, $InHg_{labile}$ and total Se in the wet brain tissue of pilot whales.

Whale	Age	Total Hg (µM)		MeHg (µM)		MeHg	$InHg_{labile}$ (µM)		$InHg_{bound}$	Se (µM)		Se:Hg
ID/Gender	year	AV	SD	AV	SD	%	AV	SD	%	AV	SD	
1 F	2.5	NA		NA		NA	NA		NA	NA		
2 F	25.5	57.6	4.36	4.35	0.183	7.53	9.85	0.032	75.4	73.0	9.8	1.3
3 F	17	NA		NA		NA	NA		NA	NA		
4 F	25*	NA		NA		NA	NA		NA	NA		
5 F	20	47.5	3.17	5.55	0.201	11.7	7.94	0.073	71.6	64.8	4.03	1.4
6 F	1*	0.902	0.123	0.615	0.018	66.3	0.215	0.018	9.87	28.6	2.61	31.7
7 F	1*	2.87	0.283	2.26	0.063	78.3	0.375	0.033	8.67	36.9	3.9	12.9
8 F	1*	0.85	0.008	0.665	0.014	76.0	0.18	0.004	2.89	21.1	3.69	24.8
9 M	6*	NA		NA		NA	NA		NA	NA		
10 F	4	3.13	0.337	2.09	0.084	66.9	0.027	0.0003	32.3	18.7	2.49	6.0
11 F	29	135	11.0	10.9	0.763	8.13	16.1	0.428	80.0	178	13.0	1.3
12 F	3	3.3	0.341	3.01	0.069	90.8	0.463	0.062	ND	26.2	1.93	7.9
13 F	9	8.47	1.11	3.68	0.332	43.5	ND		NA	30.4	3.44	3.6
14 M	2.5	2.46	0.113	1.68	0.046	68.8	0.338	0.023	17.4	22.7	0.71	9.2
15 F	35.5	NA		NA		NA	NA		NA	NA		
16 M	4	2.56	0.398	1.73	0.204	68.2	0.674	0.082	5.41	21.4	1.34	8.4
17 M	2	2.05	0.109	1.8	0.124	87.4	0.473	0.058	ND	87.9	10.1	42.9
18 F	25	NA		NA		NA	NA		NA	NA		
19 M	15	NA		NA		NA	NA		NA	NA		
20 F	28	NA		NA		NA	NA		NA	NA		
21 M	16	NA		NA		NA	NA		NA	NA		

$InHg_{labile}$ is defined as inorganic Hg susceptible to derivatisation. $InHg_{bound}$ was operationally defined as HgT - (MeHg + Hg_{labile}). Asterisk (*) indicates whales, which tooth for age determination was not available and therefore the length of the individual was used for estimation of the age. NA indicates tissues, which were not available for analysis. ND means value not defined. SD is standard of triplicate sample analysis. All concentrations are calculated in the wet weight.

Table S2 Concentrations of measured total Hg, MeHg, $InHg_{labile}$ and total Se in the wet liver tissue of pilot whales.

Whale	Age	Total Hg (mM)		MeHg (µM)		MeHg	$InHg_{labile}$ (µM)		$InHg_{bound}$	Se (mM)		Se:Hg
ID/Gender	year	AV	SD	AV	SD	%	AV	SD	%	AV	SD	
1 F	2.5	0.027	0.002	5.1	0.206	18.7	ND			0.073	0.008	2.7
2 F	25.5	1.22	0.028	30.9	2.82	2.52	ND			1.55	0.19	1.3
3 F	17	0.764	0.036	27.1	0.632	3.54	ND			0.984	0.063	1.3
4 F	25*	1.13	0.059	27.6	1.03	2.43	ND			1.32	0.044	1.2
5 F	20	0.771	0.082	16.8	0.395	2.18	ND			0.956	0.051	1.2
6 F	1*	0.005	0.0002	1.73	0.13	32.0	0.831	0.005	52.4	0.05	0.015	10.0
7 F	1*	0.007	0.001	1.93	0.068	28.2	ND			0.028	0.002	4.0
8 F	1*	0.007	0.001	2.02	0.067	28.4	ND			0.03	0.008	4.3
9 M	6*	0.219	0.015	13.7	0.492	6.27	ND			0.292	0.044	1.3
10 F	4	0.157	0.022	9.32	0.556	5.92	ND			0.211	0.017	1.3
11 F	29	2.37	0.068	29.7	0.808	1.26	141	6.73	92.8	2.57	0.361	1.1
12 F	3	0.051	0.002	7.58	0.202	14.9	ND			0.089	0.005	1.7
13 F	9	0.323	0.025	15.7	0.224	4.85	ND			0.467	0.017	1.4
14 M	2.5	0.031	0.003	4.76	0.162	15.4	ND			0.117	0.019	3.8
15 F	35.5	2.32	0.141	24.3	1.13	1.05	290	9.37	86.9	2.41	0.304	1.0
16 M	4	0.044	0.001	6.26	0.216	14.1	ND			0.081	0.007	1.8
17 M	2	0.017	0.002	3.87	0.246	22.7	ND			0.049	0.005	2.9
18 F	25	1.01	0.071	26.0	0.689	2.58	ND			1.47	0.175	1.5
19 M	15	0.324	0.013	13.1	0.633	4.04	ND			0.402	0.033	1.2
20 F	28	2.37	0.238	20.3	0.083	0.86	ND			2.68	0.694	1.1
21 M	16	1.1	0.077	17.7	0.571	1.61	ND			1.5	0.328	1.4

$InHg_{labile}$ is defined as inorganic Hg susceptible to derivatisation. $InHg_{bound}$ was operationally defined as HgT - (MeHg + Hg_{labile}). Asterisk (*) indicates whales, which tooth for age determination was not available and therefore the length of the individual was used for estimation of the age. ND indicated values, which were not defined. SD is standard of triplicate sample analysis. All concentrations are calculated in the wet weight.

Table S3 Hg L_{III} and Se K-edge speciation results generated by synchrotron XANES analysis.

	Hg speciation (%)				Se speciation (%)						
Tissue	HgSe	MeHg	MeHg-GSH	R-factor	SeCys	HgSe	$[SeCys]_2$	SeMet	Me-SeMet	$TMSe^+$	R-factor
L 6	LOD	LOD	LOD	-	12	10	15	39	22	2	0.001
L 11	88	4	9	0.004	5	66	12	9	LOD	8	0.018
B 11	79	9	12	0.004	LOD	69	14	10	LOD	7	0.004

Dash (-) indicates species below the instrumental limit of detection.

Tissue: L 6 = Liver 6; L 11 = Liver 11; B 11 = Brain 11

Table S4 Results of single-particle ICP-MS analysis, showing the largest measured size (nm) of the detected particles and the number of detected particles in the selected liver and brain tissues of pilot whales.

Tissue	Whale	Age	Gender	Max particle size (nm)		Particle concentration (particles L^{-1})	
Liver	ID	year		Hg	Se	Hg	Se
	1	3	F	247	186	8.40E+08	9.34E+07
	2	26	F	404	400	2.07E+11	1.75E+11
	3	17	F	467	538	2.89E+10	2.49E+10
	4	25*	F	346	359	2.31E+11	1.13E+11
	5	20	F	364	361	1.52E+11	1.27E+11
	6	1*	F	163	ND	6.72E+08	ND
	7	1*	F	ND	ND	ND	ND
	8	1*	F	ND	ND	ND	ND
	9	6*	M	425	339	2.10E+10	1.10E+10
	10	4	F	371	260	2.95E+10	1.52E+10
	11	29	F	363	433	2.21E+11	1.72E+11
	12	3	F	274	332	9.29E+08	4.40E+08
	13	9	F	366	325	2.26E+10	7.27E+09
	14	3	M	286	279	1.89E+09	3.87E+08
	15	36	F	405	499	2.22E+10	1.79E+10
	16	4	M	250	373	7.87E+09	3.23E+09
	17	2	M	237	208	5.00E+09	1.33E+09
	18	25	F	253	229	4.84E+10	1.47E+10
	19	15	M	358	453	1.77E+10	9.33E+09
	20	28	F	488	369	7.68E+11	5.94E+11
	21	16	M	538	559	9.84E+10	5.31E+10
**Brain**							
	2	26	F	379	399	1.03E+11	9.04E+10
	5	20	F	363	335	7.15E+10	5.00E+10
	10	4	F	149	160	2.84E+10	1.61E+10
	11	29	F	352	436	6.36E+10	5.88E+10
	13	9	F	193	235	1.95E+10	2.08E+10

Measurement was performed twice, first monitoring *m/z* ^{202}Hg and then *m/z* ^{77}Se as detection of both *m/z* simultaneously is not possible. Asterisk (*) indicates whales, which tooth for age determination was not available and therefore the length of the individual was used for estimation of the age. ND indicated values, which were not determined.
